# Clonal Propagation of *Valeriana jatamansi* Retains the Essential Oil Profile of Mother Plants: An Approach Toward Generating Homogenous Grade of Essential Oil for Industrial Use

**DOI:** 10.3389/fpls.2021.738247

**Published:** 2021-10-14

**Authors:** Rahul Dev Gautam, Ajay Kumar, Ravi Kumar, Ramesh Chauhan, Satbeer Singh, Manish Kumar, Dinesh Kumar, Ashok Kumar, Sanatsujat Singh

**Affiliations:** ^1^Agrotechnology Division, CSIR-Institute of Himalayan Bioresource Technology, Palampur, India; ^2^Academy of Scientific and Innovative Research (AcSIR), Ghaziabad, India; ^3^Chemical Technology Division, CSIR-Institute of Himalayan Bioresource Technology, Palampur, India

**Keywords:** *Valeriana jatamansi*, vegetative propagation, clone, naphthalene acetic acid, essential oil, patchouli alcohol, QPM

## Abstract

Valeriana jatamansi Jones (Syn. *V. wallichii* DC.) is an aromatic, medicinal herb used as a tranquilizer and in treating sleep disorders. Rhizome is mainly used to extract essential oil (EO) and valepotriates. High quality and economic yield of rhizomes are available in the third year of growth. Therefore, the cultivation of *V. jatamansi* is not picking up, and over-exploitation of this plant from wild habitats to meet the increasing demand of the pharmaceutical industry is the cause of threat to the genetic diversity of the species. Further, collections from the wild are heterogeneous, resulting in variable produce. The development of clonal lines can ensure uniform quality and yield of rhizome biomass. An effective clonal propagation method was standardized using different hormonal concentrations of naphthalene acetic acid (NAA) on apical shoot cuttings from the selected clone CSIR-IHBT-VJ-05 for different time durations and raised over various planting media. NAA treatment of 50 ppm concentration for 30 min was found optimum for root induction in apical shoots of *V. jatamansi*. Variations for EO composition within the clone were non-significant, while samples of the control population were variable. The best quality EO (patchouli alcohol ∼62%) was available during the third year of plant growth. A propagation technique for large-scale quality plant material (QPM) production has been standardized to reduce the stress over natural resources and promote *V. jatamansi* for use in the aromatic and pharmaceutical industry.

## Introduction

*Valeriana jatamansi* Jones, commonly known as “Indian Valerian” (English name) and “Mushkbala” (Hindi name), is a perennial aromatic medicinal herb belonging to the family Valerianaceae. It is a perennial and shade-loving plant found at elevations ranging from 1200 to 4000 m amsl in the temperate Himalayas from Kashmir to Bhutan and between 1200 and 2000 m amsl in Khasi and Jaintia hills in North East India (Anonymous, 1976). This species generally grows on shady slopes, moist places, and near streams (Jugran et al., 2013). Important constituents from rhizomes and roots of *V. jatamansi* are sesquiterpenoids (Willis et al., 2000) bakkenoloids type sesquiterpenoids (Xu et al., 2011), EO and phenolics (Bhatt et al., 2012). Iridoids from *V. jatamansi* possess anti-inflammatory and antiproliferative activity (Liu et al., 2021). EO of such herbs are the traditional natural resources which exhibit antibacterial and antioxidant properties (Mahmoudi and Nosratpour, 2013; Mahmoudi et al., 2013a,b; Jafari et al., 2014; Mohammadzadeh et al., 2018). Major interest in *Valeriana* species is focused on two groups; valepotriates and EO sesquiterpenes (Bhardwaj et al., 2021). Valepotriates chemically belong to the group of iridoids and can be extracted from the roots, rhizomes, or from whole plant (Lin et al., 2010). Extraction of iridoids is performed using powdered air dried roots with ethanol alcohol (Wang et al., 2017; Quan et al., 2019), while EO is extracted through the hydrodistillation method using Clevenger type apparatus (Thusoo et al., 2014; Jugran et al., 2020). The plant has a vital role in curing many diseases and disorders, including epilepsy, hysteria, leprosy, asthma, snakebite, and scorpion stings in Unani and Ayurvedic systems of medicine (Mukherjee, 2015). It also has important use in the preparation of *Havan* and *Dhoop* in the Indian local markets. Dua et al. (2008) reported extracts of *V. jatamansi* to be effective against the larvicidal and adulticidal activities of mosquitoes.

Due to its high-value EO, *V. jatamansi* is uprooted extensively from the wild. Increasing demand for its EO in perfumery has imposed pressure on the plant’s natural populations, leading to population loss. Many patches of its natural habitat where it was present in abundance have now disappeared (Maurya et al., 2017). Further, the quality of constituents in medicinal and aromatic plants depends on several factors, viz., age of the plants, stage of harvest, climatic variations from one location to the other, and uniformity of the population’s germplasm been raised. Conventionally, this plant is propagated through seeds and also vegetatively through the division of rhizome. Flowers of *V. jatamansi* are easily visible to pollinators due to their crowding effect favoring cross-pollination (Rather et al., 2014). In natural conditions, it is difficult to maintain uniformity in the progeny on account of uncontrolled pollination which leads to a high degree of outcrossing and development of heterogeneous populations, showing variations for chemical constituents and cause quality degradation. Poor germination of seeds (Mukherjee and Chakraborty, 2014) may affect crop stand and reduce overall production. Also, due to the slow rate of rhizome formation as crop is harvested after 2 years of transplantation (Singh et al., 2010), the vegetative propagation through the Rhizome becomes a time-consuming process. Therefore, it is vital to have elite germplasm on a large scale which can attain through an efficient clonal propagation method to maintain uniformity in the quality and yield of raw produce.

Various techniques have been studied for the propagation of *V. jatamansi*. For instance, micro-propagation in *V. jatamansi* took 5 months to develop a plant from culture initiation (Purohit et al., 2015). In *Valeriana officinalis* (a related species of *V. jatamansi*), cultured leaf explants were used for plant regeneration through direct shoot organogenesis (Abdi and Khui, 2007). Likewise, Chen et al. (2014) used leaves of *V. jatamansi* as explants for the shoot and somatic embryo induction from embryogenic callus using different concentrations of PGRs. Successful plantlet development has also been reported under *in vitro* conditions by apical and axial shoot buds of *Valeriana wallichii* (Mathur et al., 1989). Clonal propagation can be successfully achieved through optimal use of phytohormones such as naphthalene acetic acid (NAA) which are extensively used for root induction. It was used in micropropagation protocols of *Zingiber spectabile* (Faria and Illg, 1995), sugarcane (Tolera, 2016), and gerberas (Singh et al., 2016). Sarropoulou and Maloupa (2019) recorded maximum root numbers with 0.5 mg/l NAA while propagating *Satureja thymbra*. On similar lines, Firoz et al. (2015) recorded high root number, and root length in *Cucumis sativus* shoots growing on media containing 0.5 mg/L NAA. Also, rooting in *Solanum nigrum* was found better in NAA as compared to indole-acetic acid (IAA) and indole-butyric acid (IBA) (Jabeen et al., 2005). However, *in vitro* propagation methods may take 5–6 months to establish plantlets. They may not be economically feasible on a commercial scale at the farmers’ level as they involve high initial costs.

Naphthalene acetic acid has also been successfully used for inducing roots in cuttings of *Valeriana carnosa* (Nagahama et al., 2019) and other plant species such as *Hemarthria compressa* (Yan et al., 2014) and *Andrographis paniculata* (Hossain and Urbi, 2016) for achieving clonal propagation. Currently, there are no reports on the exogenous application of NAA for root induction in apical shoots of *V. jatamansi*. Therefore, the present study focuses on the potential of clonal propagation of *V. jatamansi* through the treatment of apical shoots with different concentrations and durations of NAA in various growing media (soil mixture, sand, and coco-peat) for root induction and variation among the EO composition in mother plant and clonally raised plant. Findings will help to establish a cost-effective propagation method for the multiplication of this plant species.

## Materials and Methods

### Plant Material and Growth Conditions

An elite selection ‘Him Surbhit’ (CSIR-IHBT-VJ-05), with high root production potential compared to control variety ‘Himbala’ (Anonymous, 2018), was used for clonal propagation studies suitable indigenous germplasm and is being maintained *in situ* as mother stock through division of rhizomes. The EO yield of CSIR-IHBT-VJ-05 is 0.31% as compared to 0.29% in control. CSIR-IHBT-VJ-05 (IC0630604, INGR20096) is registered with the Plant Germplasm Registration Committee, National Bureau of Plant Genetic Resources (NBPGR), ICAR, New Delhi. NAA [C_10_H_7_CH_2_CO_2_H, 2-(naphthalen-1-yl)acetic acid, molecular weight = 186.2066 g mol^–1^) was procured from Himedia (India), dissolved in ethanol, and concentrations of 50, 100, 200, and 300 ppm were prepared with distilled water. Apical shoots of CSIR-IHBT-VJ-05 with single nodes were used for raising cuttings. These cuttings are treated with different doses of NAA to evaluate the effect of NAA treatments in comparison to control (no treatment) based on the performance of apical buds for root development parameters viz., the number of roots, root length (cm), and root diameter (mm). Different planting media (soil mix, sand, and coco-peat) were used as replicates to assess the influence of the soil environment over the root development parameters.

The experiment was conducted in 2018 under controlled environmental conditions in a propagation chamber (temperature variations from 20 to 25°C) at the experimental farm of CSIR-Institute of Himalayan Bioresource Technology, Palampur (1320 m amsl; 32°68′N, 76°38′E), situated in the mid-hills of Himachal Pradesh, with humid sub-temperate climate and high mean annual rainfall (∼2500 mm). The cuttings were raised during the onset of monsoon season (end of June) in root trainers under 50% shade (using the agro-shade net) conditions, and light irrigation was done. Light irrigation was performed using watering daily for the first week of the experiment and subsequently when the soil media on the surface started drying.

A comparative study on the multiplication rate of different accessions was also undertaken in terms of new plant generation through the splitting rhizomes (after 3 years) and the number of apical shoots formed (in a year). It will help estimate quality plant material (QPM) production on a large scale to meet commercial cultivation requirements.

### Experimental Design

The experiment was carried out using factorial design to study the effect of four different hormonal (NAA) doses (ppm) and four duration of treatments (time in minutes) in comparison to control (no treatment) on clonal propagation of *V. jatamansi* replicated over different planting media viz., (a) mixture of soil, sand, and farmyard manure (1:1:1), (b) medium-fine river sand, and (c) coco-peat. The different doses and durations of NAA (α-naphthaleneacetic acid) used for the treatments are presented in Table 1.

**TABLE 1:** Different treatments (T_1_ to T_15_) of NAA doses (ppm) at different time durations.

T_01_	Control	T_02_	Control	T_03_	Control	T_04_	Control
T_1_	50 ppm NAA + 30 min	T_5_	100 ppm NAA + 30 min	T_9_	200 ppm NAA + 30 min	T_13_	300 ppm NAA + 30 min
T_2_	50 ppm NAA + 60 min	T_6_	100 ppm NAA + 60 min	T_10_	200 ppm NAA + 60 min	T_14_	300 ppm NAA + 60 min
T_3_	50 ppm NAA + 120 min	T_7_	100 ppm NAA + 120 min	T_11_	200 ppm NAA + 120 min	T_15_	300 ppm NAA + 120 min
T_4_	50 ppm NAA + 180 min	T_8_	100 ppm NAA + 180 min	T_12_	200 ppm NAA + 180 min	T_16_	300 ppm NAA + 180 min

Five cuttings were raised in each treatment. However, data recording was done on three of the most competitive rooted plants, and the mean value was generated for each treatment to satisfy the condition of homogeneity of variance. The data of root growth parameters were recorded after 28 days of planting the cuttings in the root trainers. The average number of roots per plant was counted manually, while root length (cm) and root diameter (mm) were measured using a geometrical scale and vernier calipers.

### Essential Oil Extraction

Thirty clonal plants of CSIR-IHBT-VJ-05 were raised in the field under partial shade conditions (using 50% agro-shade net) for 3 years (2018–2021) along with thirty plants of control variety ‘Himbala.’ The EO content was determined by hydrodistillation (Agnihotri et al., 2011; Thusoo et al., 2014; Kumar et al., 2020, 2021) of 500 g of cleaned fresh rhizomes of the mother plant, cloned plants and control plants during the third year in Clevenger-type apparatus for 6 h in October 2020. The EO was dried over anhydrous sodium sulfate and placed in amber-colored vials at 4°C.

### Gas Chromatography–Mass Spectrometry and Compound Identification

Gas chromatography (GC) and GC–MS analysis was performed through flame ionization detector (FID) on Shimadzu QP2010 equipped with AOC-5000 auto-injector and SH-RX-5Si/MS capillary column Shimadzu Asia pacific, United States (30 M × 0.25 mm × 0.25 μm). Helium was the carrier gas (1.28 mL min^–1^ flow rate). The oven temperature was set to 70°C for 3 min and then to 220°C for 5 min with a constant rate (4°C min). The temperatures of MS source and interface were 240 and 250°C, respectively. The mass was scanned at 70 eV from 40 to 800 amu. The sample injection was 2 μL with a split ratio of 1:10. The retention indices of compounds were calculated by using homologous series of n-alkanes (C8–C24). The RI value for each peak of GC–MS spectra was calculated and compared with Adams tabulated indexes (not exceeding ±10) (Adams, 1995) stored at the New York mass spectral (MS) library, National Institute of Standards and Technology (NIST) (Stein, 2005).

### Statistical Analysis

The experiment involved the analysis of simultaneous variations for more than one factor. Using randomized block design (RBD), we get information for a single factor at a time. The relevance of following the RBD approach depends on the condition that responds to different levels of one factor is independent of varying levels of the second factor. However, such an assumption may not work in all situations. Notably, there will be interaction among the levels of the two factors under study in the present experiment. Therefore, a practical approach is to investigate the effect of two factors together by comparing the possible combinations of different levels of both the factors in the same experiment. Accordingly, factorial analysis of variance was done to identify significant variations among the treatments (both for doses and durations) for root parameters. The significance of differences was tested by the least significant difference (LSD) at *P* ≤ 0.05. Bar plots were generated using PAST software based on doses and duration of treatments to present the trend of root parameters in response to treatment effects.

The composition of EO samples obtained from mother plants and clonal plants of CSIR-IHBT-VJ-05 was analyzed using Student’s *t*-test. Pooled standard deviations were used for the test to compare mean values for significant components of EO. Similarly, clonal plants of CSIR-IHBT-VJ-05 were compared with seed-raised plants of Himbala to understand the variations among the two diverse selections.

## Results and Discussion

### Effect of Naphthalene Acetic Acid on Rooting

**FIGURE 1:**
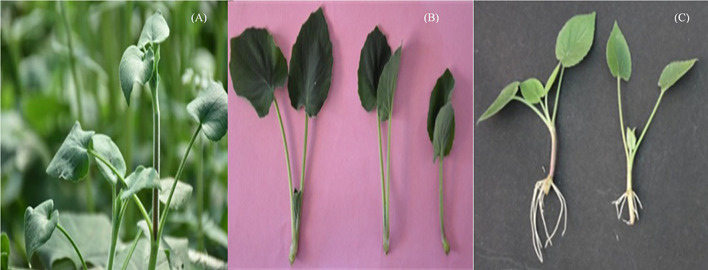
Apical buds of *Valeriana jatamansi* **(A)**, single-node cuttings **(B)**, and root formation in cuttings **(C)**, treated with NAA 50 ppm (left) and control (right).

Overall results about NAA-induced root growth in apical shoots of *V. jatamansi* indicated significant variations for different doses and durations of treatments (Figure 1). Induction of a higher number of roots in apical shoots provides easy maintenance of clonal stocks and ensures higher chances of early establishment and field survival of the rooted plants. Seed-raised nurseries of *V. jatamansi* require great care in the initial stages of growth due to small seed size and may take up to 4 months to harden the plants before transplanting to field conditions. Propagation through the division of rhizomes takes up to 3 years in *V. jatamansi* and is not feasible as rhizomes and roots are the parts utilized for the production of EO and valepotriates in this species.

**FIGURE 2:**
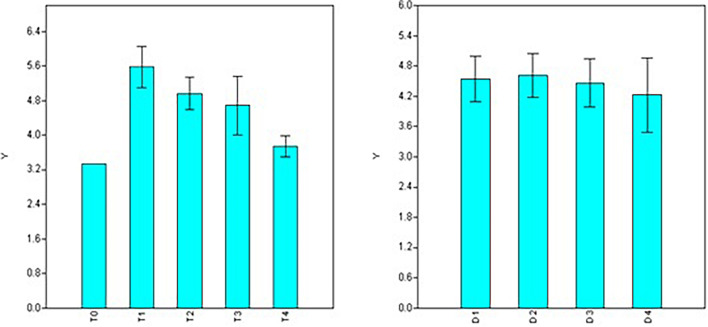
Effect of NAA doses **(left)** and durations **(right)** on number of roots formed in apical shoots of *Valeriana jatamansi*.

Observations concerning root number, length, and diameter in different NAA treatments reflected the plants’ vigor resulting from dosage and duration of treatments. Based on factorial analysis of data, significant variations were observed for doses of NAA treatments for the number of roots formed, while durations of treatment showed non-significant variations (Table 2). Maximum root number was observed using 50 ppm NAA solution (T_1_ = 5.58), and it was *at par* with the number of roots formed under 100 ppm (T_2_ = 4.96) and 200 ppm (T_3_ = 4.68) treatments (Figure 2) but significantly higher than 300 ppm treatment (T_4_ = 3.74) as well as control (T_0_ = 3.33). Number of roots formed were statistically at par for different durations of treatment (Figure 2) with maximum root numbers in 60 minutes’ duration (D_2_ = 4.61), followed by treatments for 30 min (D_1_ = 4.54), 120 min (D_3_ = 4.46), and 180 min (D_4_ = 4.23).

**TABLE 2:** Factorial analysis of data for root parameters on varying doses and durations of NAA treatments in apical cuttings of CSIR-IHBT-VJ-05 (*Valeriana jatamansi*).

Sources of variations	Degree of freedom	Mean of squares		
		Root number	Root length	Root diameter
Replications	2	7.245	51.145*	0.0933
Treatment combinations	19	6.696	5.383	0.0098
Durations	3	0.741	3.051*	0.0032*
Doses	4	17.820*	13.869*	0.0214*
Interaction	12	4.477	3.138*	0.0075*
Error	38	3.838	0.508654	0.0003

*Significant at *P* < 0.05.

**FIGURE 3:**
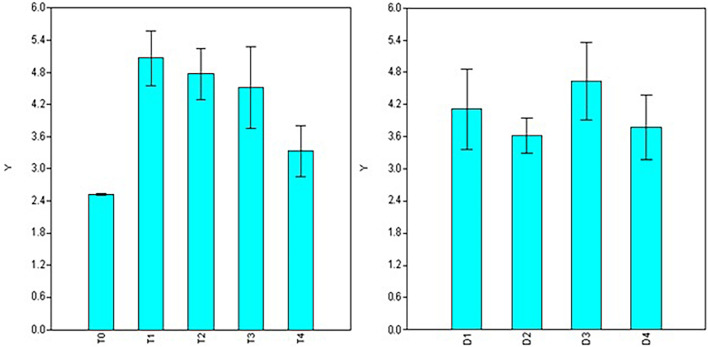
Effect of NAA doses **(left)** and durations **(right)** on root length (cm) in apical shoots of *Valeriana jatamansi*.

In the case of root length, significant variations were observed for doses, durations, and interaction between the two factors (dose × duration) involved in the treatments (Table 2). Maximum root length was observed using 50 ppm NAA solution (T_1_ = 5.06 cm) and it was at par with number of roots formed under 100 ppm treatment (T_2_ = 4.76 cm) and 200 ppm (T_3_ = 4.51 cm) but significantly higher than 300 ppm (T_4_ = 3.32 cm) treatments (Figure 3) as well as control (T_0_ = 2.51 cm). Significant variations for root length were also observed for different durations of treatment with maximum root length in duration of 120 min (D_3_ = 5.18 cm), followed by treatments for 30 min (D_1_ = 4.50 cm) and 180 min (D_4_ = 4.09 cm) while a significant decline was observed in root length in treatments of 60 min (D_2_ = 3.88 cm) duration (Figure 3).

**FIGURE 4:**
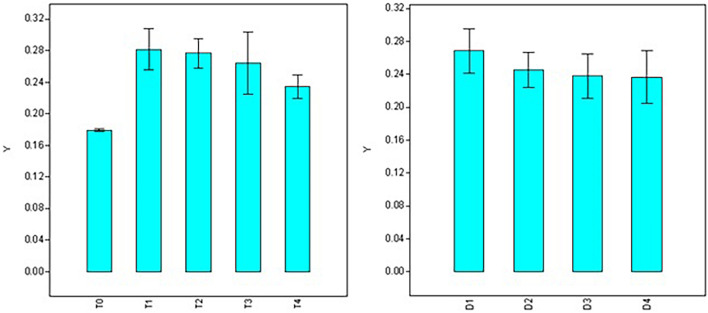
Effect of NAA doses **(left)** and durations **(right)** on root diameter (mm) in apical shoots of *Valeriana jatamansi*.

Significant variations were also observed for doses, durations, and interaction (dose × duration) when analyzing root diameter (Table 2). Maximum root diameter was observed using 50 ppm NAA solution (T_1_ = 0.282 mm) and it was at par with root diameter under 100 ppm treatment (T_2_ = 0.277 mm), 200 ppm (T_3_ = 0.264 mm), and 300 ppm (T_4_ = 0.234 mm) treatments (Figure 4), while it was significantly higher than root diameter in control (T_0_ = 0.179 mm). Root diameter was maximum in 30 min (0.266 mm) and was significantly higher than all the other durations of treatments (Figure 4) viz., 60 min (0.245 mm), 120 min (0.238 mm), and 180 min (0.237 mm).

The most important physiological effect of auxin is stimulating elongation in stems and coleoptile through cell division and cell elongation. The proper formation of adventitious roots at the base of apical shoots/stem cuttings is an essential developmental phenomenon in the growth and survival of cuttings. It involves initiating several new meristematic areas in different tissues of stem cuttings (Kaur et al., 2002). Auxin perception is believed to be mediated by receptors [Auxin Binding Protein 1 (ABP1) that physically bind auxin, allowing it to travel from outside the cell into the cell cytoplasm, where it then initiates signal transduction cascades that trigger specific physiological auxin responses. As a synthetic auxin, NAA is commonly used at a relatively low dose to elicit auxin-type responses in cell growth, cell division, fruit set, and rooting (Srivastava, 2002). Earlier studies support the observation, whereby the adventitious root production increased rapidly at lower NAA concentration, while the number of roots decreased at higher concentrations (Raju and Prasad, 2010; Sun and Hong, 2010). However, the dosage requirements of different species vary to a vast extent. Rooting in softwood cuttings of *Chimonanthus praecox* was successfully promoted using 100 mg/L NAA (Hu et al., 2020). The use of 400 mg/L NAA resulted in maximum rooting in *Couroupita guianensis* (Shekhawat and Manokari, 2016). The root development by applying NAA was more effective for root development against IBA in *V. carnosa* (Nagahama et al., 2019).

The 750 and 1000 ppm concentrations of IBA were best for rooting rosemary and geranium cuttings (Rawat et al., 2020). In our study, maximum root numbers in *V. jatamansi* were obtained at 50 ppm concentration with a decline at higher concentrations.

Contrary to dosage effects, durations of treatments do not show significant variations for root numbers. However, the analysis revealed that root length is significantly affected by the duration of treatments that peak at 120 minutes’ treatment (Figure 3). Root diameter was maximum at 30 min duration (D_1_) and declined at higher durations of treatments. The higher number of roots during the initial growth phase favors the early establishment of apical shoots/cuttings, while longer roots are prone to damage during transplantation and may turn out to be an impediment. Therefore, NAA treatment of 50 ppm concentration for 30 min may be considered optimum for root induction in apical shoots of *V. jatamansi*.

Variations were also studied for the performance of rooting parameters in different planting media. Non-significant variations were observed in terms of the number of roots formed in various planting media, and based on mean values, maximum root formation was observed in the sand (6.56) followed by coco-peat (5.93) and soil mix (5.35). Root length was significantly affected under different planting media (Table 3), with maximum length (4.52 cm) under coco-peat followed by sand (4.35 cm) and soil mix (3.24 cm). Variations observed for root diameter were non-significant under different planting media used, with maximum root diameter observed in coco-peat (0.26 mm) followed by sand (0.23 mm) and soil mix (0.23 mm).

**TABLE 3:** Analysis of variance for root parameters in apical cuttings of CSIR-IHBT-VJ-05 under different planting media.

Sources of variations	Degree of freedom	Mean of squares		
		Root number	Root length	Root diameter
Treatments	19	6.696531	5.383717	0.009822
Planting media	2	7.245362	9.664432*	0.006207
Error	38	3.838455	3.122514	0.004836

*Significant at *P* ≤ 0.05.

Sand and coco-peat have high porosity than soil mix resulting in easy elongation of roots in such media. Abigaba et al. (2020) found that sterilized sand as propagating medium for softwood cuttings of *Carissa edulis* resulted in better propagation, while Nurhayati et al. (2020) successfully propagated *Cinnamomum zeylanicum* plants in the coco-peat medium. Overall, in the present study, both sand and coco-peat gave better propagation results. However, significant variations in comparison to soil mix were not evident except for the root length parameter.

### Essential Oil Analysis

A comparative study of EO was performed to understand the variations of EO metabolites in mother plants, clonally propagated CSIR-IHBT-VJ-05 plants, and control plants (Himbala seed raised plants) an open-pollinated plant population variety. Overall, 38 compounds were identified in the EO using GC–MS analysis, which accounted for 98.1–100.0% of the total EO profile of *V. jatamansi* samples (Table 4).

**TABLE 4:** Essential oil composition of mother plants, clonally propagated plants of CSIR-IHBT-VJ-05, and seed raised plants of Himbala.

S. No.	Compound name	RT	RI	RI Lit.	CSIR-IHBT-VJ-05-1	CSIR-IHBT-VJ-05-2	CSIR-IHBT-VJ-05-3	C- CSIR-IHBT-VJ-05-1	C- CSIR-IHBT-VJ-05-2	C- CSIR-IHBT-VJ-05-3	HB1	HB2	HB3	Range (%)
1	3-Methyl-pentanoic acid	5.778	–	–	0.54	–	0.43	0.32	0.67	0.35	–	–	–	0.32–0.67
2	α*-*Pinene	5.396	938	932	–	–	–	–	–	–	0.64	0.74	0.67	0.64–0.74
3	α*-*Fenchyl acetate	16.947	1283	–	0.56	0.53	0.52	0.53	0.53	0.54	–	–	–	0.52–0.56
4	Bicyclo[2.2.1] heptane, 7,7-dimethyl-2-methylene	5.774	–	–	–	–	–	–	–	–	1.65	1.49	1.4	1.4–1.65
5	Limonene	8.063	–	–	–	–	–	–	–	–	0.36	–	–	0.36
6	2-Isopropyl-5-methyl-1-methoxybenzene	14.795	–	–	–	–	–	–	–	–	0.6	0.62	0.6	0.6–0.62
7	4,7-Methanoazulene, 1,2,3,4,5,6,7,8-octahydro-1,4,9,9- tetramethyl-, [1S-(1.alpha.,4.alpha.)]	20.497	–	–	–	–	–	–	–	–	–	1	–	1
8	β-Patchoulene	20.498	1385	1381	1.3	1.26	1.28	1.25	1.28	1.26	0.99	–	0.95	0.95–1.3
9	Cyclohexane, 1-ethenyl-1-methyl-2,4-bis(1-methylethenyl)-, [1S-(1.alpha.,2.beta.,4.beta.)]-	20.641	–	–	1.55	1.53	1.52	1.49	1.52	1.51	0.97	–	0.92	0.92–1.55
10	Santalene	21.620	1419	1417	0.6	0.58	0.66	0.66	0.63	0.65	7.02	6.58	6.29	0.58–7.02
11	Calarene	22.060	–	–	1.21	1.22	1.24	–	1.22	1.2	–	–	–	1.2–1.24
12	Trans-α*-*bergamotene	22.090	–	–	–	–	–	–	–	–	4.07	4.01	3.89	3.89–4.07
13	α-Guaiene	22.186	1438	1439	3.06	3.03	3	2.99	3.02	3.02	2.92	2.81	2.74	2.74–3.06
14	Cis-caryophyllene	22.469	–	–	–	–	–	–	–	–	–	16.2	15.4	15.4–16.2
15	Trans-caryophyllene	22.453	1447	–	1.25	1.22	1.2	1.22	1.2	1.19	19.13	1.9	–	1.19–19.13
16	Seychellene	22.672	1451	1446	4.79	4.94	4.73	4.79	4.72	4.73	–	5.05	–	4.72–5.05
17	α*-*Humulene	22.868	1457	1454	2.09	2.16	2.09	2.12	2.08	2.11	1.85	1.75	1.72	1.72–2.16
18	α*-*Patchoulene	23.079	1463	1456	2.24	2.34	2.22	2.26	2.23	2.24	1.96	1.87	1.8	1.8–2.34
19	Patchoulene	23.183	1468	–	0.31	0.3	–	0.29	–	0.31	5.4	–	4.95	0.29–5.4
20	γ-Curcumene	23.542	–	–	–	–	–	–	–	–	7.29	6.97	6.49	6.49–7.29
21	Benzene, 1-(1,5-dimethyl-4-hexenyl)-4-methyl- (CAS) ar-curcumene	23.640	–	–	–	–	–	–	–	–	3.25	3.17	2.98	2.98–3.25
22	Zingiberene	24.074	–	–	–	–	–	–	–	–	–	0.56	–	0.56–0.56
23	Eudesma-4(14),11-diene	23.957	–	–	0.39	0.24	0.43	–	–	0.37	–	–	–	0.24–0.43
24	α-Selinene	24.191	1495	1498	1.34	1.45	1.39	1.34	1.37	1.34	0.83	0.96	0.93	0.83–1.45
25	β-Elemene	24.279	–	–	1.31	1.26	1.33	1.31	1.2	1.3	–	0.94	–	0.94–1.33
26	(Z)-Bisabolene α-DB5-1655	24.280	–	–	–	–	–	–	–	–	–	–	1.8	1.8
27	δ-Guaiene	24.369	–	–	6.58	–	6.61	–	6.65	–	–	5.33	–	5.33–6.65
28	Azulene	24.375	–	–	–	6.86	–	6.64	–	6.56	5.62	–	5.04	5.04–6.86
29	Cyclohexene, 1-methyl-4-(5-methyl-1-methylene-4-hexenyl)-, (S)-	24.525	–	–	–	–	–	–	–	–	–	–	1.08	1.08
30	β-Bisabolene	24.519	–	–	–	–	–	–	–	–	0.96	1.13	–	0.96–1.13
31	(-)-α*-*Panasinsen	24.947	1520	–	1.67	1.56	1.77	1.74	1.74	1.76	1.09	1.05	1.02	1.02–1.77
32	Kessane DB5-1710	25.257	1530	1529	0.59	0.6	0.61	0.58	0.59	0.58	0.5	0.6	0.57	0.5–0.61
33	1-Naphthalenol, decahydro-1,4a-dimethyl-7-(1-methylethylidene)-, [1R-(1.alpha.,4a.beta.,8a. alpha)]	26.793	–	–	–	–	–	2.99	2.28	3.46	–	–	–	2.28–3.46
34	Juniper camphor	26.658	1580	–	2.87	2.3	3.21	1.04	1.82	–	–	–	–	1.04–3.21
35	Veridiflorol	26.629	1587	–	0.6	0.59	1.21	–	–	1.18	–	–	–	0.59–1.21
36	Cis-α*-*bisabolene	25.543	–	–	–	–	–	–	–	–	0.77	0.74	0.8	0.74–0.8
37	Patchouli alcohol	29.705	1656	1658	62.92	63.39	62.35	62.75	62.28	62.12	30.66	32.93	36.47	30.67–63.39
38	Longifolenaldehyde	31.076	1690	–	1.84	1.84	1.83	1.79	1.81	1.81	1.47	1.6	1.49	1.47–1.84
	Total				99.61	99.2	99.63	98.1	98.84	99.59	100.0	100.0	100.0	98.1–100.00

*RT is retention time, RI is retention indices on SH-RX-5Si/MS capillary column, RI Lit. is retention indices from literature.*

The EO metabolites such as 3-methyl-pentanoic acid (0.32–0.67%), α-fenchyl acetate (0.32–0.67%), calarene (0.32–0.67%), eudesma (0.32–0.67%), naphthalenone (0.32–0.67%), juniper camphor (0.32–0.67%), and veridiflorol (0.32–0.67%) were detected in samples of mother plant and clones of CSIR-IHBT-VJ-05 but absent in samples of Himbala, while the β-patchoulene (0.95–1.3%), cyclohexane1-ethenyl-1-methyl-2,4-bis (1-methylethenyl) (0.92–1.55%), trans-caryophyllene (1.19–19.13%), seychellene (4.72–5.05%), and β-elemene (0.94–1.33%) were present among samples of mother plant and clones of CSIR-IHBT-VJ-05 but not detected in all samples of control. On the other hand, α-pinene (0.64–0.74%), bicycloheptane (1.4–1.65%), limonene (0.36%), 2 isopropyl methoxy benzene (0.6–0.62%), methanoazulene (1.0%), trans-α-berganotene (3.89–4.07%), cis-caryophyllene (15.4–16.2%), γ-curcumene (6.49–7.29%), benzene curcumene (2.98–3.25%), zingiberene (0.56%), α-bisabolene (1.8%), cyclohexene (1.08%), β-bisabolene (0.96–1.13%), and cis-α-bisabolene (0.74–0.8%) were detected exclusively in samples of Himbala, while these were absent in samples of clone CSIR-IHBT-VJ-05. Some of the compounds viz., patchoulene (0.29–5.4%), δ-guaiene (5.33–6.65%), and azulene (5.04–6.86%) were identified exclusively in Himbala and CSIR-IHBT-VJ-05, but not in all the samples analyzed, while 1-naphthalenol, decahydro-1,4a-dimethyl-7-(1-methylethylidene)-, [1R-(1.α.,4a.β.,8a.α.)] (2.28–3.46%) was identified in clones of CSIR-IHBT-VJ-05.

**FIGURE 5:**
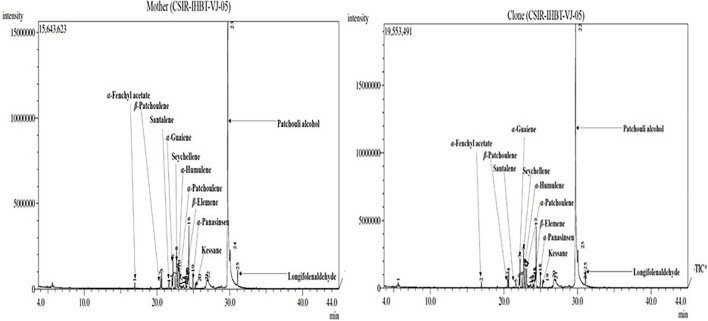
Chromatograms of essential oil sample of mother and clonal plants of CSIR-IHBT-VJ-05.

Fifteen compounds were detected of EO of mother and clonal plants ofCSIR-IHBT-VJ-05, which accounted for 86.39% of EO (Table 5). Based on the *t*-test using pooled standard deviations, variations for all these components were statistically non-significant among the mother plants and clonal plants of CSIR-IHBT-VJ-05 except for longifolenaldehyde. Based on mean values the components of EO in both mother and clonal plants of CSIR-IHBT-VJ-05 were patchouli alcohol (62.89% in mother plant/62.38% in clones), seychellene (4.82/4.74%), α-guaiene (3.03/3.01%), α-patchoulene (2.27/2.24%), α-humulene (2.11/2.10%), longifolenaldehyde (1.84/1.80%), α-panasinsen (1.67/1.75%), cyclohexane (1.53/1.51%), α-selinene (1.39/1.35%), β-elemene (1.3/1.27%), β-patchoulene (1.28/1.26%), trans-caryophyllene (1.22/1.20%), santalene (0.61/0.65%), kessane (0.60/0.58%), and α-fenchyl acetate (0.54/0.53%), respectively (Figure 5).

**TABLE 5:** Variations of essential oil compounds in mother plant and vegetatively propagated clone of CSIR-IHBT-VJ-05.

Compound name	CSIR-IHBT-VJ-05 (mother plants)			CSIR-IHBT-VJ-05 (clonal plants)			
	Mean	Variance	SD	Mean	Variance	SD	*t*-value
α*-*Fenchyl acetate	0.54	0.000433	0.020817	0.53	0.00003	0.005774	0.307056
β-Patchoulene	1.28	0.0004	0.02	1.26	0.00023	0.015275	1.15728
Cyclohexane	1.53	0.000233	0.015275	1.51	0.00023	0.015275	2.138012
Santalene	0.61	0.001733	0.041633	0.65	0.00023	0.015275	1.434699
α*-*Guaiene	3.03	0.0009	0.03	3.01	0.00030	0.017321	1.035238
Trans-caryophyllene	1.22	0.000633	0.025166	1.20	0.00023	0.015275	1.211334
Seychellene	4.82	0.0117	0.108167	4.75	0.00143	0.037859	1.230074
α*-*Humulene	2.11	0.001633	0.040415	2.10	0.00043	0.020817	0.400025
α*-*Patchoulene	2.27	0.004133	0.064291	2.24	0.00023	0.015275	0.718303
α-Selinene	1.39	0.003033	0.055076	1.35	0.00030	0.017321	1.466108
β-Elemene	1.30	0.0013	0.036056	1.27	0.00370	0.060828	0.75846
(-)-.α*-*Panasinsen	1.67	0.011033	0.10504	1.75	0.00013	0.011547	1.680741
Kessane	0.60	0.0001	0.01	0.58	0.00003	0.005774	2.588096
Patchouli alcohol	62.89	0.271233	0.520801	62.38	0.10723	0.327465	1.453394
Longifolenaldehyde	1.84	0.00003	0.005774	1.80	0.00013	0.011547	4.713872*

*Significant at *P* < 0.05 (t-tab. = 4.303), where SD is standard deviation. t-tab, t-tabulated value.

Overall, patchouli alcohol is the major component of *V. jatamansi* EO which forms the largest fraction (∼ 62%). Earlier studies on EO have revealed populations which exhibited about 63.7 and 65.04% of patchouli alcohol, respectively (Singh et al., 2013; Raina and Negi, 2015). The present study on GC–MS analysis reveals that clonal plants of a high yielding selection can produce a uniform grade of EO, which is a primary requirement of the aroma industry.

**FIGURE 6:**
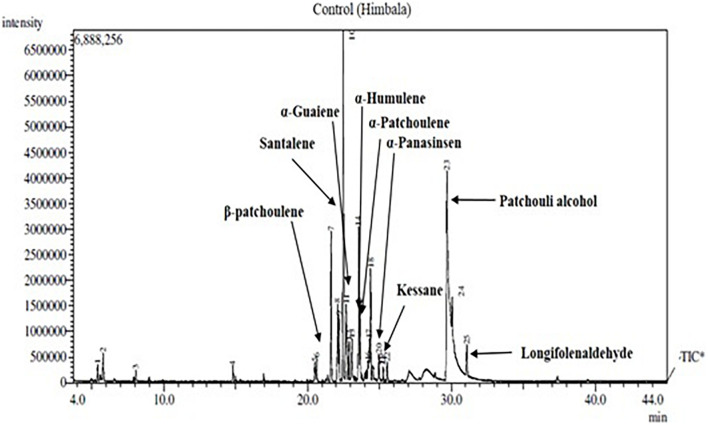
Chromatograms of essential oil sample of Himbala.

Nine components of EO were detected in mother and clonal plants of CSIR-IHBT-VJ-05 and EO from plants of ‘Himbala’ (Table 6 and Figure 6). Based on *t*-test using pooled standard deviations, significant variations were obtained for patchouli alcohol (62.38% in clone of VJ05/33.35% in seed raised plants of Himbala), α-patchoulene (2.24/1.88%), α-humulene (2.10/1.77%), longifolenaldehyde (1.80/1.52%), α-panasinsen (1.75/1.05%), α-selinene (1.35–0.91%), and santalene (0.65/6.63%). Only α-guaiene (3.01/2.82%) and kessane (0.64–0.92%) were found statistically non-significant components among the EO samples of clonal plants of CSIR-IHBT-VJ-05 and Himbala. Samples of Himbala plants varied from CSIR-IHBT-VJ-05 and each other for different EO components (Table 6), as is evident from high variance and standard deviation values CSIR-IHBT-VJ-05 clone.

**TABLE 6:** Variation of essential oil compounds in a vegetatively propagated clone of CSIR-IHBT-VJ-05 and seed raised plants of Himbala.

Compound name	CSIR-IHBT-VJ-05 (clonal plants)			Himbala seed raised plants			
	Mean	Variance	SD	Mean	Variance	SD	*t*-Value
Santalene	0.65	0.00023	0.0152	6.63	0.13510	0.3675	38.281*
α*-*Guaiene	3.01	0.00030	0.0173	2.82	0.00823	0.0907	4.231
α*-*Humulene	2.10	0.00043	0.0208	1.77	0.00463	0.0680	9.093*
α*-*Patchoulene	2.24	0.00023	0.0152	1.88	0.00643	0.0802	9.405*
α*-*Selinene	1.35	0.00030	0.0173	0.91	0.00463	0.0680	12.717*
(-)-.α.-Panasinsen	1.75	0.00013	0.0115	1.05	0.00123	0.0351	36.391*
Kessane	0.58	0.00003	0.0057	0.56	0.00263	0.0513	1.144
Patchouli alcohol	62.38	0.10723	0.3274	33.35	8.57343	2.9280	21.832*
Longifolenaldehyde	1.80	0.00013	0.0115	1.52	0.00490	0.07	8.510*

*Significant at *P* < 0.05 (t-tab. = 4.303), where SD is standard deviation. t-tab, t-tabulated value.

Variations in EO profile of clonal line CSIR-IHBT-VJ-05 and open-pollinated plant population of variety ‘Himbala’ reveal chemotypic distinction among the two. CSIR-IHBT-VJ-05 (collection from Salooni region in district Chamba, Himachal Pradesh) and Himbala (selection from Palampur region, district Kangra, Himachal Pradesh) have diverse characteristics geographic origin and accordingly are expected to differ for their EO composition. Earlier studies based on the EO composition of *V. jatamansi* from Himalayan regions of India (Singh et al., 2010, 2013; Verma et al., 2013; Raina and Negi, 2015) confirm chemotypic and seasonal variations among accessions collected from different regions. The pre-flowering stage is reported to have high concentrations of phenols, flavonoids, and higher antioxidant activity while the decrease in concentration of monoterpenes and sesquiterpenes with increase in oxygenated sesquiterpenes due to qualitative and quantitative variation in EO was observed among different phenological stages across altitude gradient (Jugran et al., 2020). In our study, EO was extracted during month of October from all the samples studied to ensure homogeneity. Open-pollinated varieties derived through mass selection tend to be more variable on phenotypic and chemotypic basis with time, owing to random mating among the individuals of the population leading to generation of new variations on account of genetic reconstitution resulting in heterogenous genetic makeup. The flower size of *V. jatamansi* is small with plants having either hermaphrodite or pistillate flowers. The high degree of entomophilous pollination is evident during the flowering stage in *V. jatamansi*, ensuring cross-fertilization. However, experiments on plants with hermaphrodite flowers in isolation also result in some seed-set due to selfing. Studies on genetic diversity and population structure of natural populations from the western Himalayan region using amplified fragment length polymorphism (Rajkumar et al., 2011) suggest high genetic variation (93%) within populations. Natural populations during evolution acquire such adaptive floral mechanisms to ensure cross-fertilization and achieve fitness of the populations. However, from a cultivation point of view, clonal selection presents the advantage of producing a homogenous genetic background and thereby control at least one of the factors (uniform planting material) contributing to the quality of produce in this commercially important herb.

### Estimates for Quality Plant Material Production

The multiplication rate in *V. jatamansi* through rhizome splits per plant is 17.07 plants after 3 years of the growth period, whereas 22.47 apical shoots are available per plant right in the first year of growth (Table 7).

**TABLE 7:** Multiplication rate of plants raised through rhizome split and apical shoots method.

S. No.	Selections	Rhizome split (3rd year)	Apical shoots (1st year)
1	CSIR-IHBT-VJ-01	20.00	23.00
2	CSIR-IHBT-VJ-02	8.67	20.67
3	CSIR-IHBT-VJ-03	26.67	22.00
4	CSIR-IHBT-VJ-04	17.00	20.67
5	CSIR-IHBT-VJ-05	28.33	26.67
6	CSIR-IHBT-VJ-06	8.67	18.33
7	CSIR-IHBT-VJ-07	14.00	25.00
8	CSIR-IHBT-VJ-08	24.67	28.33
9	CSIR-IHBT-VJ-09	14.67	20.67
10	Himbala	8.00	19.33
	Mean	17.07	22.47

**FIGURE 7:**
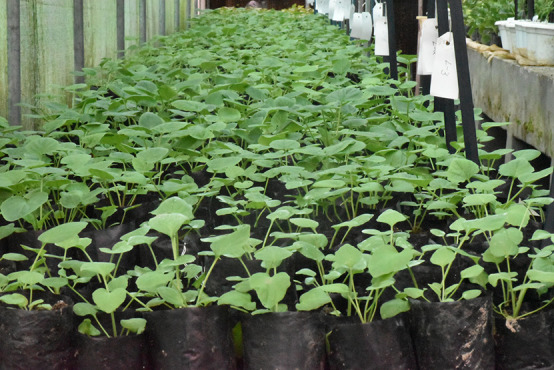
Clonally raised plant nursery of *Valeriana jatamansi*.

With a start from 10 plants, three multiplication cycles are required using apical shoots to produce around 80,000 plants to cover a 1-ha cultivation area. About 74,074 plants ha^–1^ are required for plantation at 30 × 45 cm spacing, which maximizes underground mass (Mukherjee, 2015). No mortality of clonally raised plants using apical shoots was observed, and its multiplication can be undertaken very well using sand or coco-peat as the planting media. Plants can be grown from apical shoots in root trainers, and space requirements for raising 80,000 plants is 167 m^2^, which can be reduced to half, one-third, or even further following two-tier, three-tier, and multi-tier planting systems. Thus, large-scale QPM production of *V. jatamansi* can be ensured through the improvised clonal propagation method even under the limitation of mother plants’ improved selections (Figure 7). Also, the EO obtained from clonal plants is of uniform quality, similar to the mother plant’s chemical profile, leading to higher returns.

## Conclusion

Based on the results, significant variations among treatments of NAA were observed for root growth parameters in apical shoots of *V. jatamansi*. Factorial analysis of variance revealed a substantial influence of NAA dosage on the number of roots formed, while treatment durations were non-significant. On the other hand, both root length and diameter were significantly affected due to doses, durations, and interaction between them (dose × duration). NAA treatment of 50 ppm concentration for 30 min was found optimal for root induction in apical shoots of *V. jatamansi*. Comparisons of EO from mother plants, clonally propagated plants of CSIR-IHBT-VJ-05, and control plants using GC–MS analysis revealed statistically non-significant variations for major oil components among the mother plants and clonal plants of CSIR-IHBT-VJ-05 highlighting the potential of clonal plants to produce the uniform grade of EO. Selection CSIR-IHBT-VJ-05 was a unique chemotype rich in patchouli alcohol (∼62%) and distinct from the existing open-pollinated variety Himbala. This selection has potential utility in breeding to improve rhizome biomass and EO quality of *V. jatamansi*. NAA-induced rooting in apical cuttings of *V. jatamansi* led to the establishment of a simple and fast propagation method that can be utilized for efficient large-scale QPM production to ensure uniformity of produce.

## Data Availability Statement

The original contributions presented in the study are included in the article/supplementary material, further inquiries can be directed to the corresponding authors.

## Author Contributions

RG: clonal propagation experiment. AjK: performed oil extraction. RK: data recording. RC: implemented standard agronomic practices in experiments. StS: data analysis. MK: chemical profiling of essential oils. DK: chemical evaluation of essential oil and manuscript editing. AsK: germplasm collection from diverse locations and manuscript editing. SnS: planning and monitoring of experiment, important suggestions, and the manuscript writing. All authors contributed to the article and approved the submitted version.

## Conflict of Interest

The authors declare that the research was conducted in the absence of any commercial or financial relationships that could be construed as a potential conflict of interest.

## Publisher’s Note

All claims expressed in this article are solely those of the authors and do not necessarily represent those of their affiliated organizations, or those of the publisher, the editors and the reviewers. Any product that may be evaluated in this article, or claim that may be made by its manufacturer, is not guaranteed or endorsed by the publisher.
